# Phytochemistry, Toxicology, and Pharmacological Properties of *Origanum elongatum*

**DOI:** 10.1155/2021/6658593

**Published:** 2021-06-17

**Authors:** Balahbib Abdelaali, Naoual El Menyiy, Nasreddine El Omari, Taoufiq Benali, Fatima-Ezzahrae Guaouguaou, Najoua Salhi, Hanae Naceiri Mrabti, Abdelhakim Bouyahya

**Affiliations:** ^1^Laboratory of Biodiversity, Ecology and Genome, Mohammed V University in Rabat, Rabat, Morocco; ^2^Laboratory of Physiology, Pharmacology and Environmental Health, University Sidi Mohamed Ben Abdellah, Fez, Morocco; ^3^Laboratory of Histology, Embryology, and Cytogenetic, Mohammed V University in Rabat, Rabat, Morocco; ^4^Environment and Health Team, Polydisciplinary Faculty of Safi, Cadi Ayyad University, Marrakech, Morocco; ^5^Mohammed V University in Rabat, LPCMIO, Materials Science Center (MSC), Ecole Normale Supérieure, Rabat, Morocco; ^6^Laboratory of Pharmacology and Toxicology, Faculty of Medicine and Pharmacy, Mohammed V University in Rabat, BP 6203, Rabat, Morocco; ^7^Laboratory of Human Pathologies Biology, Department of Biology, Mohammed V University in Rabat, Rabat, Morocco

## Abstract

*Origanum elongatum* L. is an endemic aromatic and medicinal plant. This work reports previous studies on *O. elongatum* concerning its taxonomy, botanical description, geographical distribution, bioactive compounds, toxicology, and biological effects. Chemical analyses showed that *O. elongatum* contains different chemical compounds, in particular volatile compounds. Pharmacological investigations showed that volatile compounds and extracts from *O. elongatum* exhibit different pharmacological properties, such as antibacterial, antifungal, antiviral, antioxidant, vasodilator, corrosion inhibitor, and hepatoprotective effects. Moreover, toxicological reports revealed the safety of this species. The pharmacological effects of *O. elongatum* could be correlated with the main compounds, which exhibit different pharmacological properties with numerous mechanism insights.

## 1. Introduction

*Origanum elongatum* (Bonnet) Emberger et Maire is an endemic aromatic and medicinal species of Morocco. It is a medicinal plant of the Lamiaceae family, a perennial herb of the *Origanum* genus. It is distributed in the wild species and is limited to the northeast (NE) of Morocco and extends from the Middle Atlas to the Rif Mountains ranges, mainly at high altitude on the mountains (the mountain of Tazekka and the mountain of Bouyablane).

Phytochemical investigations showed that *O. elongatum* contains several classes of bioactive compounds, including terpenoids, flavonoids, oxygenated compounds, hydrocarbon compounds, and phenolic compounds [1–8]. The main volatile compounds of this species are carvacrol, thymol, linalool, and limonene. Chemical analysis showed that the chemical composition is different between several published studies depending on the plant part and the collection site.

Pharmacological reports showed that extracts and essential oils (EOs) of *O. elongatum* exhibited various biological activities such as antibacterial [3, 4, 6, 9–11], antifungal [3, 12], antiparasitic [5, 13–16], antiviral [6], antioxidant [1], vasodilator [10, 17], corrosion inhibitor [18], and hepatoprotective effects [19]. These biological effects are certainly due to the chemical composition of *O. elongatum*, in particular the main compounds such as carvacrol, linalool, and thymol. Indeed, literature reports revealed that these compounds possess several pharmacological effects. Moreover, the toxicological investigations showed that *O. elongatum* caused a slight change in behavior with loss of appetite and temporary sedation without any change in pathophysiological and neurological activity and LD_50_ was greater than 3000 mg/kg [19].

This review is designed to explore all previous studies on *O. elongatum* L. in terms of taxonomy, botanical description, geographic distribution, ethnobotanical prospecting, and toxicology and all the investigations on the biological activities of the different parts of this plant, and we will summarize the list of all phytochemicals isolated and identified from the extracts or EOs of this plant. This work aims to provide a scientific basis for further studies and the development of medicinal agents from *O. elongatum*.

## 2. Research Methodology

The collection of data about *Origanum elongatum* concerned its botanical description, taxonomy, destruction, phytochemistry, and biological properties. Numerous databases such as Google Scholar, Web of Science, Scopus, ScienceDirect, SpringerLink, Wiley Online, SciFinder, and PubMed were consulted to collect publications about *O. elongatum*. The collected data have covered all years. The collected articles were organized in tables, analyzed, and highlighted in this review according to each field. The chemical compounds of O. elongatum were PubChem database and their chemical structures were drawn using ChemDraw Pro 8.0 software.

## 3. Results and Discussion

### 3.1. Botanical Description

*Origanum elongatum* (Bonnet) Emberger et Maire is an endemic species of Morocco. It is a woody perennial, which stems up to 90 cm. Its stems are erect, light or dark brown (purplish) and at the bases hirsute (hairs *c*. 1.5 mm long), otherwise, glabrous, and often glaucous leaves. Branches of the first order are present, in the upper 1/3−1/2 of the stems, up to 15 pairs per stem, 4 cm long; branches of the second order sometimes present; those of the third order seldom so. All branches entirely or for the greater part consist of spikes. Leaves up to 30 pairs per stem are shortly petiolate in the lower part to subsessile in the upper part (petioles up to 5 mm long), ovate or oval, margins entire, tops obtuse, 10 mm long, 8 mm wide, somewhat leathery, light green or purplish, often glaucous, glabrescent (pilose to glabrous; hairs *c*. 1.2 mm long), sessile glands up to 1600 per cm^2^. Spikes are very loose and tenuous, 40 mm long, *c*. 3 mm wide. Bracts are 10 pairs per spike, ± lanceolate, tops acute, 3 mm long, 1 mm wide, glabrous or pilosellous, green, often glaucous. Flowers are subsessile. Calyces are 3.5 mm long, outside glabrous or pilosellous; teeth 1 mm long. Corollas are 6 mm long, pink, outside pilosellous; upper lips divided, for *c*. 1/5, into 2, *c*. 0.3 mm long lobes; lower lips divided, for *c*. 3/5, into 3, somewhat unequal, 1 mm long lobes. Staminal filaments are up to 2 and 3.5 mm long. Styles are up to 8 mm long [20].

### 3.2. Taxonomy and Geographic Distribution

*Origanum* is derived from two Greek words, “oros” which means mountain and “ganos” which means shine; this word would mean “ornament of the mountains” [21]. The appearance of the French term was in the 13th century, European (*Origanum* sp.) and Mexican (Lippia sp.) “oregano.” The name “Oregano” is commonly used around the world to define a spicy aroma and flavor [22].

The genus *Origanum* belongs to the Lamiaceae family and the Nepetoideae subfamily. The taxonomic point of view was completely revised by Dr. JH Ietswaart in 1980 [20]. In this work, Ietswaart divided the genus into 3 groups, 10 sections, grouping in total 38 species (one with 6 subspecies and another with 3 varieties), and 17 hybrids. This classification was based on the morphological characters of the plant (length of the stem, number of branches, and shape of the leaves) [20].

The flora of Morocco includes five taxa of *Origanum*, of which two (*O. compactum* and *O. vulgare* subsp. virens) are considered to be Ibero-Moroccan taxa and the other three taxa (*O. elongatum*, *O. grosii*, and *O. fontqueri*) are endemic in Morocco [2]. Due to their very similar morphologies, these three Moroccan steno-endemics are subject to taxonomic confusion. According to some databases [23], *O. grosii* and *O. × fontqueri* are considered to be synonyms of *O. elongatum*. However, Ietswaart [20] described *O. grosii* and *O. elongatum* as two different *Origanum* species according to their morphological characters, the length of the stems, ears, and leaves of *O. Elongatum* being larger than those of *O. Grosii*, but with *O. grosii* having a longer and wider bract than *O. elongatum*, while the hybrid, *O. × fontqueri*, is not described in the Ietswaart classification [20]. On the other hand, *O. elongatum* and *O. grosii* show obvious morphological differences from *O. compactum* (section Prolaticorolla Ietsw.) and from *O. vulgare* subsp. virens (section *Origanum* L.).

*Origanum elongatum* (Bonnet) Emb. & Maire belongs to the Elongatispica section (Section 7) of group C [20]. It is recognized in Morocco by its common Arabic name “Zaatar.” The geographical distribution of the wild species is limited to the NE of Morocco and extends from the Middle Atlas to the Rif Mountains ranges, mainly at high altitude on the mountains (the mountain of Tazekka and the mountain of Bouyablane) [20].

### 3.3. Ecological Factors

The wild species of *O. elongatum* grows at altitudes between 400 and 1500 m [24]; it abounds in open forests, rockeries, and mountain matorrals, on siliceous substrates and deep and well-drained soils. It is characterized by a fairly significant bioclimatic plasticity ranging from semiarid to per humid. The most favorable vegetation stages for this species are the thermo-Mediterranean and the meso-Mediterranean [25]. These oregano flowers from June to October [20] are known for their white inflorescence attached to vertical stems [24]. The abundance of their inflorescences, their lightness, and the sequence of flowering provide an ornamental interest to this species [20]. *O. elongatum* grows readily in temperate continental climates and grows rapidly, but with limited development in size. However, harvesting is possible in the first year but with a low yield of dry matter [7]. The germination of *O. elongatum* seeds is extremely affected by abiotic factors, such as temperature, salinity stress, and pH. Thus, a temperature of 20°C, a pH of 6, and a salinity of 1 g/L constitute the optimal conditions for germination of this species [26].

### 3.4. Phytochemistry

**Figure 1:**
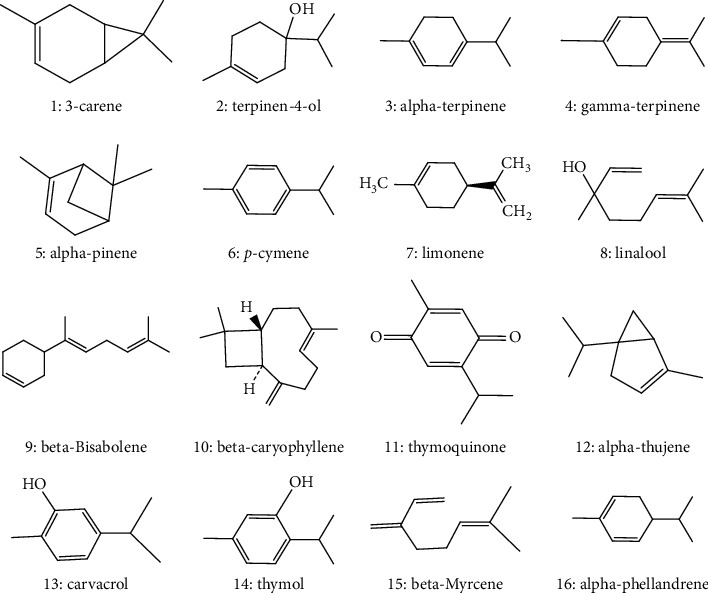
Chemical structures of terpenoids identified in *O. elongatum* EOs.

The phytochemical analysis of *O. elongatum* extracts and EOs revealed the presence of a set of compounds, which are summarized in Table 1. Terpenoids were among the chemical classes dominating in *O. elongatum* EOs (Figure 1).

The methanol extract and the ethyl acetate extract from the leaves of *O. elongatum* are rich in phenolic compounds. The total phenol content of these extracts is 153.22 ± 2.67 mg GAE/g of methanol extract and 130 ± 3.0567 mg GAE/g of ethyl acetate extract. However, the flavonoid content is 4.83 ± 0.72 mgEQ/g of ethyl acetate extract and 5.02 ± 0.26 mgEQ/g of methanol extract [17].

The analysis by gas chromatography of *O. elongatum* EO from Morocco shows that it consists of 11 compounds, of which 3 are in the majority; carvacrol (60.42%), *p*-cymene (13.9%), and *γ*-terpinene (9.4%) [1].

In the same country (Morocco), 28 compounds were identified in *O. elongatum* EO, including carvacrol, thymol, and *p*-cymene, constituting the majority compounds. Additionally, limonene, thymoquinone, and thymohydroquinone have been reported in some EOs [2].

The main compounds identified in *O. elongatum* EO are carvacrol (63.06%), *γ*-terpinene (15.99%), *p*-cymene (9.51%), and other compounds, with appreciable percentages such as *α*-phellandrene, caryophyllene, and *α*-pinene [3].

On the other hand, a previous study [4] showed that the chemical profile of *O. elongatum* EO shows the predominance of oxygenated compounds (65.14%), followed by hydrocarbon compounds (28.02%), knowing that thymol is the major compound with 63.44%. These results confirm the findings of [5], which showed that the main constituents found in the aerial parts of *O. elongatum* EO are carvacrol (67.34%), *γ*-terpinene (3.29%), *p*-cymene (3.62%), and thymol (1.79%). However, in 2013, Moussaoui et al. [6] showed that the main constituents identified in the *O. elongatum* EO are carvacrol (40.12%), thymol (14.24%), *p*-cymene (16.19%), and *γ*-terpinene (13.48%).

Chromatographic analysis of the seeds of *O. elongatum* EO revealed the richness of the chemical composition predominated by carvacrol with a percentage of 79.2%, followed by *γ*-terpinene (3.7%), *p*-cymene (5.2%), and linalool (2.4%) [7, 8].

### 3.5. Pharmacological Studies

**Figure 2:**
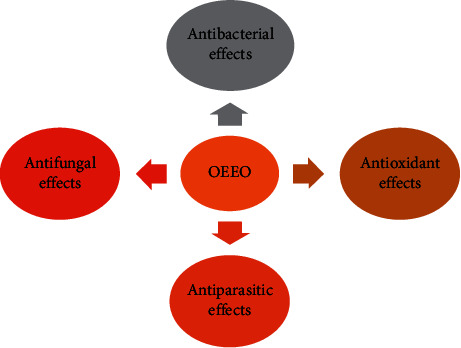
Pharmacological properties of *O. elongatum*.

EOs and extracts from *O. elongatum* showed different pharmacological properties such as antibacterial, antiparasitic, anticancer, and antioxidant effects (Figure 2). In the following part, all of these biological activities will be discussed.

### 3.6. Antibacterial Activity

Several studies showed the antibacterial efficacy of different EOs or extracts from different *O. elongatum* parts [3, 4, 6, 9–11].  Table 2 summarizes all the studies evaluating the antimicrobial activity of *O. elongatum* extracts and EOs.

Bouharb et al. [9] evaluated *in vitro* the antibacterial activity of two extracts (aqueous and ethanolic) of *O. elongatum* leaves, from the Zerhoun region (central Morocco), on the growth of six strains of *Pseudomonas aeruginosa* (P, P3, P65, P381, P2, and P5), using the agar diffusion method and the broth macrodilution method of the active extract. The screening test revealed that *O elongatum* aqueous extract was more active than the ethanolic extract, with zones of inhibition ranging from 9.33 to 11.83 and 8.33 to 11 mm, respectively. Douhri et al. [19] also studied the antibacterial activity *in vitro* of ethanolic extracts of *O. elongatum* leaves. The results showed a very important antimicrobial effect against *Escherichia coli* (30.33 ± 2.51 mm). Moreover, El Harsal and colleagues evaluated the antimicrobial activity of the volatile fractions extracted from the hydrosol (DVF) and of EOs from *O. elongatum* aerial parts growing in northern Morocco against four bacterial strains; *E. coli* ATCC 25922, *E. coli* K12, *S. aureus* ATCC 25923, and *B. subtilis* DCM 6633. They found that the antibacterial effect of DVF was significantly higher than that of total EO. The DVF was active against all the studied bacteria; the strongest effect was observed on *B. subtilis* DCM 6633 with a large inhibition zone (41.0 ± 2.6 mm). Also, the total EO of *O. elongatum* was highly active against *S. aureus* ATCC 25923, *B. subtilis* DCM 6633, and *E. coli* ATCC 25922, with inhibition zones ranging from 21.3 to 24.6 mm, while a moderate effect was observed against *E. coli* K12 [4].

In another study, the *O. elongatum* EO extracted from flowering tops and that extracted from leaves were tested against five microorganisms (*Salmonella* S64, *Salmonella* CECT 915, *Listeria monocytogenes* CECT4031, *L. monocytogenes* L23, and *E. coli* O157 : H7 CECT4267), using the disk-diffusion assay and the microtitration assays. Additionally, the EOs showed the highest activities against the microorganisms tested, in particular against *Salmonella* and *L. monocytogenes* with zones of inhibition varying between 21.67 ± 0.58 mm and 34.33 ± 4.04 mm. The moderate activity was recorded against *E. coli*, with zones of inhibition from 14.33 ± 2.52 to 19.67 ± 1.15 mm [6]. Furthermore, a concentration of 0.06% of *O. elongatum* EO, extracted from flowering tops, showed a significant increase in the growth of total mesophilic aerobic flora (FMAT) [10]. In addition, the antibacterial activity of the *O. elongatum* EO (aerial part flowering) was tested against *Staphylococcus aureus*, *P. aeruginosa*, and *E. coli*. Therefore, an important inhibitory activity against all the strains tested was observed, with an inhibition diameter between 9.33 and 35.67 mm and high efficacy against *E. coli* and *S. aureus* [3].

### 3.7. Antifungal Activity

*O. elongatum* is one of the plants with antifungal properties [3, 12]. Indeed, several studies have evaluated these properties in *O. elongatum* EOs (Table 3). The antifungal activity of the essential oil of *O. elongatum* aerial parts was tested and evaluated by the microdilution method against three strains of fungi: *Candida*, *Aspergillus*, and *Rhizopus* [12]. Therefore, all *Candida* strains showed marked sensitivity to the essential oil. The *Rhizopus* strain was less sensitive, whereas for *Aspergillus*, this oil showed an effect only on tree strains. In another study, the antifungal activity of *O. elongatum* EO was evaluated by the agar plug diffusion method, which consequently showed promising results against *Aspergillus brasiliensis* (no measurable zone of inhibition) and *Candida albicans* (33.67 ± 0.33 mm) [3].

### 3.8. Antiparasitic Activity

Many species of the genus *Origanum* have shown antiparasitic activities [13–15]. Moreover, the antiparasitic effect of *O. elongatum* was reported by several investigators (Table 4) [5, 16]. In 2017, Ramzi and collaborators [5] tested the acaricidal activity of the EOs of *O. elongatum* leaves on the *Varroa* mite. Therefore, these plant-derived EOs showed certain effectiveness against *Varroa*. Besides, the antiparasitic effect of *O. elongatum* EO was evaluated in experimental animals (female Wistar rats) infected with 6 *Anisakis* larvae using the gastric catheter method [16]. This technique was also used to administer *O. elongatum* (46.9 mg/0.5 mL of olive oil). Consequently, an EO activity against larva L3 of *Anisakis pegreffii* was observed; moreover, significant alterations of the esophageal region and the cuticle were detected in a large number of recovered larvae.

### 3.9. Antiviral Activity

The *O. elongatum* EOs, cultivated in northern Morocco, were studied for the inactivation of *Murine norovirus* (MNV-1) (Table 4), which is a human norovirus surrogate. Interestingly, the EOs from leaves and flowering tops showed antiviral activities of 0.87-0.50 log_10_TCID_50_/mL reduction and 0.75 log_10_TCID_50_/mL reduction, respectively [6].

### 3.10. Other Biological Activities

Besides the antiparasitic and antiviral activities, the antioxidant effect of endemic *O. elongatum* was examined in the Rif in northern of Morocco (Table 4) [1, 17]. The authors evaluated the vasodilator activity of *O. elongatum leaves*, extracted by methanol and ethyl acetate on a Wistar rat mesenteric vascular bed precontracted with norepinephrine. Measurement of the perfusion pressure of the rat mesenteric bed revealed that the methanolic extracts (PP = 50 mmHg) gave more active substances than the leaves of *O. elongatum* extracted in ethyl acetate (PP = 20 mmHg) [17]. Furthermore, the food conservation aspect of flowering tops of the studied plant on fresh pomegranate juice was demonstrated [10]. The findings demonstrated that the EOs combined with heat reduce the growth of natural flora presented in the pomegranate juice and thus improve the juice conservation process, while nutritional and organoleptic qualities were also preserved. Moreover, the impact against corrosion inhibition of *O. elongatum* leaves and flowers, using a mixture of methanol/chloroform, was deeply elaborated by applying the electrochemical impedance spectroscopy (EIS), mass loss method, and adsorption isotherms method [18]. According to their electrochemical parameters measurement, it was shown that the use of extracts decreased the corrosion potential (Ecorr), which ranged from −399.446 mV/ESC to −365.607 mV/ESC. A similar decrease was observed with corrosion current (jcorr). Nonetheless, a significant increase of charge transfer resistance (Rct) was noted, with the increase of the OEE concentration (the ability of layer protection from corrosion on the mild steel) [18]. Another important impact of *O. elongatum* was surveyed by Douhri et al. [19].

Scientists have shown the hepatoprotective effect of methanolic leaf extracts of this species at different doses against the toxicity induced by carbon tetrachloride (CCl_4_) in rats. The biochemical examination of serum hepatic biomarkers showed a significant decrease in serum aminotransferase levels, the canalicular enzyme, and alkaline phosphatase and reduction in the destruction of hepatic cell architecture at the dose of 2000 mg/kg/d [19].

### 3.11. Toxicology

*O. elongatum* is an aromatic plant well known for its flavor and widely consumed in Morocco as a condiment and food preservative [24]. From a toxicological point of view, only one study investigated the toxicological properties of *O. elongatum* extract by evaluating acute oral toxicity [19]. The results showed a slight change in behavior with loss of appetite and temporary sedation without any change in pathophysiological and neurological activity with an LD_50_ greater than 3000 mg/kg. Jenner et al. [27] also reported the same signs in rats when testing carvacrol, the major component of *O. elongatum*, in an acute oral toxicity test. The LD_50_ in this test was 810 mg/kg, suggesting that carvacrol is the active ingredient responsible for the behavioral change caused by this plant [27].

## 4. Biological Mechanism Insights into *O. elongatum* Main Compounds

The potent anti-inflammatory activity of the extracts encouraged the authors to isolate the main compounds (thymol, carvacrol, limonene, *α*-pinene, and linalool), which might be responsible for the anti-inflammatory effect. The reported studies showed that thymol inhibits inducible lymphocyte proliferation [28], reduces edema and leukocyte influx to injured areas [29], and induces membrane stabilization (84.11%) in human red blood cell membrane stabilization assay [30]. Moreover, this molecule had inhibitory effects on various inflammatory mediators such as IL-1*β*, IL-6, TNF-*α*, and TNF-*β* [31]. It also decreased c-Fos, NFAT-1, and NFAT-2 expression, with inhibition of inducible phospho-SAPK/JNK and phospho-STAT3 levels [32]. Molecular investigations showed that thymol also inhibits TLR4 upregulation and suppresses IKK, i*κ*b*α*, and p65 phosphorylation [33]. Additionally, carvacrol has also shown an anti-inflammatory effect [34]. This compound was reported to be able to inhibit the production of PGE2 (inflammatory mediator catalyzed by COX1) and suppress COX-2 promoter activity by activating PPAR*α* and PPAR*γ*. It also reduced the expression of LPS-induced COX-2 mRNA and protein, suggesting that the action of carvacrol on COX-2 is mediated through its agonistic effect on PPAR*γ* [35]. Moreover, Yoon et al. [36] showed that limonene decreased the production of proinflammatory cytokines and inflammatory mediators in macrophages by the inhibition of *LPS*-induced *NO* and *PGE2*, which decreased *iNOS* and *COX-2* expression [36]. This monoterpene also exhibited an anti-inflammatory effect *via* the inhibition of some signaling pathways leading to the inflammatory process in leukemia (HL-60) cell lines, such as ROS, monocyte chemoattractant protein-1 (MCP-1), NF-*κ*B, and p38 mitogen-activated protein kinase (MAPK) [37]. On the other hand, *α*-pinene exhibited potent activity in the inflammatory process and neuropathic pain [38]. It inhibited ear edema at 0.15 h (120–135% vs. 175%), paw edema at 12 h (146 ± 6%), and a decrease in COX-2 (115 ± 74% vs. 202 ± 20%) [39] and reduced the level of IL-6 in the hippocampus, cortex, and striatum [40]. In addition, linalool is another monoterpene, which also exhibited an anti-inflammatory effect. This compound significantly reduced hypersensitivity and paw edema at doses of 50 and 200 mg/kg of carrageenan-induced edema model in rats [41, 42].

Several studies showed the antidiabetic effect of different compounds identified in *O. elongatum.* The authors showed that carvacrol exhibits antidiabetic effects via several mechanisms such as reduction in blood glucose and insulin levels, decrease in (HOMA-IR) index, and decrease in the expressions of the mRNA of gluconeogenic genes, PEPCK, and G6Pase [43]. Additionally, carvacrol may also decrease glucose levels by lowering HbA_1c_, G6Pase, and FBPase activities. It also promoted the activities of glucokinase and glucose-6-phosphate dehydrogenase in the liver and protected pancreatic islets [44]. This monoterpene inhibited the activity of *α*-amylase (IC_50_ = 152.3 ± 1.21 *μ*g mL^−1^), *α*-glucosidase (IC_50_ = 94.02 ± 0.78 *μ*g mL^−1^) [45], and *β*-galactosidase [46]. On the other hand, limonene ameliorates glucose homeostasis by increasing hepatic glycogen with a decrease in plasma glucose and HbA_1c_ levels and suppresses the activities of gluconeogenic enzymes (G6Pase and FBPase) [47]. Moreover, it ameliorates the reduction of FBG level and glucose tolerance along with the activation of PPAR*α* signaling [48]. In addition, using two different cell lines, C2C12 skeletal muscle cells [49] and 3T3-L1 preadipocytes [50], limonene has been shown to improve glucose absorption by increasing phosphorylation of activated protein kinase B (Akt) and promoting p38 mitogen-activated protein kinase (p38MAPK) [50].

Furthermore, linalool also showed an antidiabetic effect [51, 52]. This compound was reported to be able to decrease blood glucose, HbA_1c_, fructosamine, IL-6 and TNF-*α*, and area under the curve of (AUC_glucose_) glucose value and increase insulin level [52]. On the other hand, thymol was also able to treat hyperglycemia by normalizing blood sugar, plasma insulin, HbA_1c_, and insulin resistance index [53]. Rhayour et al. [54] investigated the expression levels of genes involved in insulin transcription in STZ-induced diabetic rats and reported an increase in expression of the *Mafa* and *Pdx1* genes.

**Figure 3:**
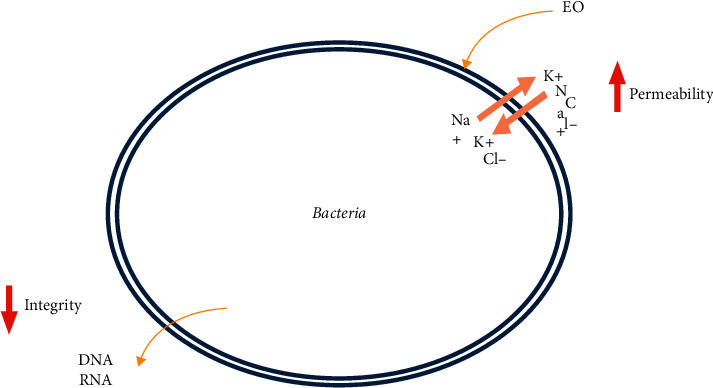
Antibacterial action of *O. elongatum* bioactive compounds.

The major compounds of this plant are limonene, linalool, carvacrol, and thymol. In fact, these molecules have shown in some studies a significant antibacterial power [55–57]. Rhayour et al. [58] examined the mechanism of action of thymol on bacteria *E. coli* and *Bacillus subtilis* as the model of Gram-positive and Gram-negative bacteria. This action was demonstrated by the release of absorbent substances at 260 nm. This release of substances associated with rapid bacterial mortality could be the consequence of lesions on the envelopes induced by antibacterial agents (Figure 3). Another study [59] showed that carvacrol affects cell membranes of bacteria by changing the composition of fatty acids, which subsequently affects the fluidity and permeability of the membrane. On the other hand, several studies have indicated that linalool alters normal cell morphology, destroys the cell wall and cell membrane, inhibits the growth of *P. aeruginosa*, and even leads to its death [60]. However, the mechanism of action of limonene against cytoplasmic membranes of microorganisms results in a loss of membrane integrity (Figure 3), inhibition of respiratory enzymes, and dissipation of the proton motive force [61].

Numerous studies have been published on the anticancer activity of the main compounds of oregano EOs such as limonene, carvacrol, and thymol [62–64]. Islam et al. [63] determined that the mechanism of action of thymol in a cancer cell caused severe DNA damage through several mechanisms (e.g., ROS induction and subsequent increase in oxidative stress and/or mitochondrial dysfunction or nuclear factor of activated T-cells (NFAT-2) pathway), which eventually upregulates Bax/Bcl-2 protein expression and results in the cytochrome- (cyto-) c release from the mitochondria (intrinsic pathway). In another work, carvacrol treatment induced cell apoptosis, possibly through the activation of the mitochondrial apoptotic, MAPK, and PI3K/Akt signaling pathways. Taken together, our results indicate that carvacrol might be a promising natural product in the management of colon cancer [65].

## 5. Conclusion

Morocco is a country rich in plant resources with a specific diversity of medicinal plants used in the treatment and prevention of several illnesses. This study provides evidence that the Moroccan *O. elongatum* L. species possesses active principles that exhibit marked therapeutic effects confirming and justifying the popular uses of these plants to treat certain diseases as antibacterial, antifungal, antiviral, antioxidant, vasodilator, corrosion inhibitor, and hepatoprotective agents. The current study represents useful documentation that can provide sufficient support for clinical trials of *O. elongatum* L. Although preliminary studies have confirmed their therapeutic effect, further investigations should be carried out, in particular, to ensure the safety of the treatment.

## Figures and Tables

**Table 1:** Chemical composition of extracts and essential oils of *O. elongatum*.

Country	Part	Extracts/essential oils	Compounds groups	Compounds	References
Morocco	Leaves	Ethyl acetate and methanol extracts	Total polyphenols	—	[17]
			Flavonoids	—	
Morocco	Leaves and flowering tops	Essential oil	Terpenoids	*α*-Thujene, *β*-myrcene, *p*-cymene, *γ*-terpinene, linalool, terpinene-4-ol, thymol, carvacrol, *β*-caryophyllene, *β*-bisabolene, and caryophyllene oxide	[1]
Morocco	Aerial parts	Essential oil	Terpenoids	Carvacrol, thymol, *p*-cymene, *α*-terpinene, limonene, thymoquinone, and thymohydroquinone	[2]
Morocco	Aerial parts	Essential oil	Terpenoids	Carvacrol, *γ*-terpinene, *p*-cymene, *α*-phellandrene, caryophyllene, 3-carene, and *α*-pinene	[3]
Morocco	Aerial parts	Essential oil	Terpenoids	Thymol, *γ*-terpinene, and *p*-cymene	[4]
Morocco	Aerial parts	Essential oil	Terpenoids	Carvacrol, thymol, *γ*-terpinene, and *p*-cymene	[5]
Morocco	Leaves and flowering tops	Essential oil	Terpenoids	Carvacrol, thymol, *γ*-terpinene, and *p*-cymene	[6]
Morocco	Seeds	Essential oil	Terpenoids	Carvacrol, thymol, *γ*-terpinene, *p*-cymene, and linalool	[8]
Morocco	Seeds	Essential oil	Terpenoids	Carvacrol, *p*-cymene, *γ*-terpinene, and linalool	[7]

**Table 2:** Antibacterial effects of *O. elongatum*.

Use part	Extracts	Used method	Tested strains	Key results	References
Leaves	Ethanolic extract	Method of diffusion in solid medium Macromethod of dilution in liquid medium	P	—	[1]
			P3	Ф = 10 ± 0.8 mm	
			P65	Ф = 11 ± 0.8 mm	
			P381	Ф = 8.5 ± 0.4 mm	
			P2	Ф = 9 ± 0.8 mm	
			P5	Ф = 8.33 ± 0.8 mm	
Leaves	Aqueous extract		P	Ф = 11.66 ± 0.47 mm	
			P3	Ф = 11 ± 0.95 mm	
			P65	Ф = 11.83 ± 0.23 mm	
			P381	Ф = 9.33 ± 1.24 mm	
			P2	Ф = 10 ± 0 mm	
			P5	Ф = 9.5 ± 0.4 mm	

Aerial parts	Essential oil	Agar diffusion methods, broth microdilution assay	*Staphylococcus aureus*	Ф = 35.67 ± 0.66 mm	[3]
				MIC = 0.03%	
				MMC = 0.13%	
			*Escherichia coli*	Ф = 26.33 ± 1.66 mm	
				MIC = 0.03%	
				MMC = 0.03%	
			*Pseudomonas aeruginosa*	Ф = 9.33 ± 0.66 mm	
				MIC = 0.5%	
				MMC = 0.5%	

Leaves	Ethanolic extract	Agar-well diffusion assay	*Escherichia coli*	Ф = 30.33 ± 2.51 mm	[11]

Leaves	Essential oil	Disk-diffusion assay Microtitration method	*Salmonella* CECT 915	Ф = 34.33 ± 4.04 mm	[6]
				MIC = 0.0625%	
				MBC = 0.0625%	
			*Salmonella* S64	Ф = 28.17 ± 1.61 mm	
				MIC = 0.0625%	
				MBC = 0.125%	
			*E. coli* O157 : H7 CECT4267	Ф = 19.67 ± 1.15 mm	
				MIC = 0.25%	
				MBC = 0.5%	
			*L. monocytogenes* CECT4031	Ф = 34.00 ± 0.00 mm	
				MIC = 0.125%	
				MBC = 0.0625%	
			*L. monocytogenes* L23	Ф = 31.00 ± 3.46 mm	
				MIC = 0.5%	
				MBC = 0.5%	

Flowering tops	Essential oil	Disk-diffusion assay Microtitration method	*Salmonella* CECT 915	Ф = 31.50 ± 2.78 mm	[6]
				MIC = 0.0625	
				MBC = 0.125%	
			*Salmonella* S64	Ф = 28.17 ± 1.61 mm	
				MIC = 0.0625%	
				MBC = 0.125%	
			*E. coli* O157 : H7 CECT4267	Ф = 18.00 ± 0.00 mm	
				MIC = 0.25%	
				MBC = 0.5%	
			*L. monocytogenes* CECT4031	Ф = 29.00 ± 1.73 mm	
				MIC = 0.125%	
				MBC = 0.25%	
			*L. monocytogenes* L23	Ф = 31.00 ± 3.46 mm	
				MIC = 0.5%	
				MBC = 0.5%	

Flowering tops	Essential oil		Total mesophilic aerobic flora (FMAT)	Significant effect on microbial growth	[10]

Aerial parts	Essential oil	Agar-well diffusion method Microdilution assay	*E. coli* ATCC 25922	Ф = 21.33 ± 0.57 mm	[4]
				MIC = 0.5%	
				MBC < 1%	
			*E. coli* K12	Ф = 16.00 ± 1.00 mm	
				MIC = 0.25%	
				MBC = 0.5%	
			*B. subtilis* DCM 6633	Ф = 24.66 ± 1.52 mm	
				MIC = 0.5%	
				MBC = 0.5%	
			*S. aureus* ATCC 25923	Ф = 27.00 ± 1.73 mm	
				MIC = 0.125%	
				MBC = 0.125%	

Aerial parts	Dissolved volatile fraction	Agar-well diffusion method Microdilution assay	*E. coli* ATCC 25922	Ф = 28.33 ± 0.57 mm	[4]
				MIC = 0.125%	
				MBC = 0.25%	
			*E. coli* K12	Ф = 17.00 ± 1.73 mm	
				MIC = 0.125%	
				MBC = 0.125%	
			*B. subtilis* DCM 6633	Ф = 41.00 ± 2.64 mm	
				MIC = 0.0625%	
				MBC = 0.0625%	
			*S. aureus* ATCC 25923	Ф = 30.00 ± 2.00 mm	
				MIC = 0.0312%	
				MBC = 0.0312%	

**Table 3:** Antifungal activity of *O. elongatum*.

Use part	Extracts	Used method	Tested strains	Key results	References
Aerial parts	Essential oil	Agar plug diffusion method	*Candida albicans*	Ф = 33.67 ± 0.33 mm	[3]
			*Aspergillus brasiliensis*	No measurable zone of inhibition	

Aerial parts	Essential oil	Microdilution method	*Candida*	Sensitive to the essential oil	[12]
			*Aspergillus*	Susceptible to the oil	
			*Rhizopus*	Moderately susceptible to the oil	

**Table 4:** Other activities of *O. elongatum*.

Activities	Use part	Extracts	Experimental approach	Key results	References
Antiviral	Leaves	Essential oil	Cytopathogenic murine norovirus (MNV-1) RAW 264.7 cells	0.37 log_10_TCID_50_/ml reductions	[6]
	Flowering tops	Essential oil	Cytopathogenic murine norovirus (MNV-1) RAW 264.7 cells	0.75 log_10_TCID_50_/ml reductions	[6]

Antiparasitic	Leaves	Essential oil	Colonies of *Apis mellifera* bees Efficacy against *Varroa* mite in beehives	Significant increase in mite drop All *Varroa* mites died	[5]
	Leaves	Essential oil	Larva L3 of *Anisakis pegreffii* isolated from the host *Scomber japonicas* and *Trachurus trachurus* Female Wistar rats infected with 6 *Anisakis* larvae by gastric catheter Administration of *O. elongatum* (46.9 mg/0.5 mL of olive oil)	Significant larvicidal activity Significant alterations in the esophageal region and cuticle detected in a large number of recovered larvae	[16]

Antioxidant	Leaves	Essential oil	EC_50_ = 1.20 g of extract/g DPPH		[1]

Vasodilatory activity	Leaves	Methanol extract	Perfusion pressure (PP) of the mesenteric bed of the rat Synthesis inhibitor endothelial vasodilators factors Vasoconstrictor *α*-mimetic: phenylephrine (PHE) Difference between the blood pressure before injection and blood pressure after injection	Vasodilatory activity (PP = 50 mmHg)	[17]

Vasodilatory activity	Leaves	Ethyl acetate extract	Perfusion pressure (PP) of the mesenteric bed of the rat Synthesis inhibitor endothelial vasodilators factors Vasoconstrictor *α*-mimetic: phenylephrine (PHE) Difference between the blood pressure before injection and blood pressure after injection	Vasodilatory activity (PP = 20 mmHg)	[17]

Effect on pomegranate juice quality	Flowering tops	Essential oil	pH variation Determination of total sugars Growth of natural flora in the pomegranate juice	Improved the juice conservation process while preserving the nutritional and organoleptic qualities	[10]

Corrosion inhibition	Leaves and flowers	Methanol/chloroform extract	Corrosion current density (jcorr) Electrochemical measurements Electrochemical impedance spectroscopy (EIS) Mass loss method Adsorption isotherms	Corrosion potential (ecorr) decreased from −399.446 mV/ESC to −365.607 mV/ESC Significant decrease in corrosion current (jcorr) Increased the charge transfer resistance (Rct) with increased OEE concentration Ability of OEE to act as a protective layer against corrosion on mild steel	[18]

Hepatoprotective effect against carbon tetrachloride (CCl4)	Leaves	Methanol extract	Single-dose intraperitoneal injection of carbon tetrachloride (CCl4) (0.6 ml/kg) induced hepatotoxicity in rats Rats treated orally by gavage four doses of 250, 500, 1000, and 2000 mg/kg body weight	Significant (*P* < 0.0001) decrease in serum aminotransferase levels and canalicular enzyme ALP reduced the architectural destruction cells	[19]

## Data Availability

The data used to support the findings of this study are included within the article.
